# Task-shifting the provision of DMPA-SC in the DR Congo: Perspectives from two different groups of providers^☆^^☆☆^

**DOI:** 10.1016/j.contraception.2018.07.002

**Published:** 2018-11

**Authors:** Julie H. Hernandez, Pierre Akilimali, Annie Glover, Rebecca Emel, Albert Mwembo, Jane Bertrand

**Affiliations:** a Global Health Management and Policy, Tulane University School of Public Health and Tropical Medicine, 1440 Canal St, Suite 1900, New Orleans, LA 70112, USA; b Kinshasa School of Public Health, University of Kinshasa, Kinshasa, Democratic Republic of the Congo; c École de Santé Publique de Lubumbashi, University of Lubumbashi, Lubumbashi, Democratic Republic of the Congo

**Keywords:** Family planning, Democratic Republic of Congo, Task-shifting, DMPA-SC, Providers

## Abstract

**Objectives:**

To document the experience of three groups of Family Planning service providers participating in task-shifting for the provision of subcutaneous depot-medroxyprogesterone acetate (DMPA-SC) at the community level in the DRC.

**Study design:**

This article compares results from interviews with DMPA-SC providers in two separate pilot studies: 1) 53 medical and nursing school students teaching women how to self-inject (2016–2017); and 2) 34 lay community health workers providing DMPA-SC in rural areas of Lualaba (2017). All providers gave information on socio-demographic characteristics, recruitment,) training, supervision, experience and satisfaction with the provision of DMPA-SC. The paper examines variations in responses from the different provider cadres.

**Results:**

Despite substantive variations in provider profiles in terms of age, educational and marital status, reported levels of satisfaction with offering DMPA-SC in the community were consistently high. Over 90% of all providers declared being comfortable or very comfortable interacting with FP clients, and more than three quarters of them were very comfortable performing an injection. Over 90% of Lualaba providers and over 80% of student providers gave correct responses to DMPA-SC protocol questions regarding referral of clients to facilities and side-effects management. The vast majority declared being (very) satisfied with their experience providing DMPA-SC.

**Conclusions:**

Providers with and without a clinical background, when properly trained and supervised, can provide DMPA-SC at the community level in both urban and rural settings of the DRC. Support strategies from the Family Planning environment (continuous contraceptive supplies and adequate referral system to fixed facilities) are key to engaging community health workers and sustainably leveraging task-shifting opportunities.

**Implication statement:**

This study provides additional evidence on the acceptability and feasibility of task-shifting in relation to DMCP-SC and supports further scale-up efforts.

## 1 Introduction

In the Democratic Republic of Congo, the task-shifting of family planning (FP) services is not a new idea [1], but the commitment of the country to the FP2020 initiative since 2012 has boosted the interest for community-based provision of contraceptives as a way to decrease unmet need^1^ for FP. The use of Community-Based Distributors (CBDs) for contraceptive provision is a key element of the National Strategic Plan for Family Planning [2]. However, existing national guidelines typically precluded non-medically trained CBDs from providing clinical contraceptives such as injectables, implants and IUDs. In a context where the first two are among women's preferred methods [3], FP partners and the Ministry of Health compromised in 2015 to test the feasibility and acceptability of allowing medical and nursing students (hereafter, the “students”) to provide subcutaneous depot-medroxyprogesterone acetate (DMPA-SC) at the community level [4], [8].

The success of the 2015 pilot created opportunities for new task-shifting initiatives, which included the use of a similar cadre of students to teach women in their community to self-inject the contraceptive^2^ in Kinshasa, and the provision of DMPA-SC by non-clinically trained Community Health Workers, known locally as *“Relais Communautaires”* (hereafter, “CHWs”) in the mostly rural province of Lualaba.

While there is a growing body of evidence concerning the acceptability of DMPA-SC provision at the community level from the perspective of FP clients [5], [6], fewer studies have been conducted from the perspective of the CBDs themselves. Following on efforts to compare acceptability of community-based DMPA-SC provision by different cadres of clinical and non-clinical personnel in Uganda and Senegal [7], the objective of this paper is to evaluate the experience of two different groups of providers (medical and nursing students in Kinshasa and CHWs with a non-medical profile in Lualaba), as they remain a key component for the success of any task-shifting program.

Results from this analysis are important in informing future initiatives, as low- and middle-income countries seek to expand access to DMPA-SC.

## 2 Materials and methods

### 2.1 Pilot interventions

Two separate pilot studies were conducted in DRC with a common objective to assess the feasibility and acceptability of community-based provision strategies for DMPA-SC. The first pilot used 53 students to teach women in target neighborhoods to self-inject the contraceptive. The providers were recruited by faculty in large, government-accredited medical and nursing schools among students who had completed clinical modules on injection, volunteered to participate and successfully completed a 6 days training on providing the full range of contraceptives authorized for community-based provision (condoms, pills, cyclebeads and DMPA-SC), referring women to facilities for additional FP services and training interested clients to self-inject DMPA-SC. The schools, supported by the project partners, provided the students with vests, hats, contraceptives (including 15 to 20 doses of DMPA-SC), printed check-lists and job aids, and reporting forms.

The community-based events, known as « campaign days » in DRC, were advertised throughout the communities and typically took place in the courtyard of health facilities to provide a modicum of privacy from the busy streets. Women who came to the campaign days expected the methods to be free and were first counseled as a group by students who presented the benefits of FP and the methods they could provide. The students then proceeded with the clients to chairs set around the courtyard (or in small consultation rooms) for individual counseling. After the client chose her method and was screened for eligibility, the student recorded the service data. For DMPA-SC clients, the student offered to train them to self-inject and if the woman was interested, demonstrated on a mannequin. Correct self-injection techniques were validated using a checklist and women declared skilled by the students were invited to self-inject and come back 3 months later for re-injection. The same campaign days were organized then, and women were again invited to practice self-injection. At the end of this second round, students provided the women they deemed competent with three doses of DMPA-SC to re-inject at home at 6, 9 and 12 months. The entire process was supervised by professors from the medical and nursing schools and personnel from the National Program for Reproductive Health (PNSR), who observed at least one interaction per student and provided immediate feedback to improve service quality. Visual privacy inside the courtyard of the health facility was non-existent, a common issue with healthcare provision in DRC, but the students were able to maintain audio privacy. In addition, although the facility was compensated to provide its courtyard, its staff did not participate in the event. Women who wanted an implant or IUDs were referred to the facility and had to pay the regular user fees. The students were not remunerated since these events are considered part of their practicum in the nursing schools curriculum. Each of the six target sites (3 per target Health Zone in urban and rural areas of Kinshasa) organized three campaign days per round (initial injection and 3 months follow-up), for a total of 36 campaign days.

The second pilot took place in two rural Health Zones of the Lualaba province. Community-health workers (CHWs) already involved in community immunization or nutrition programs were invited to participate in the pilot and trained for 6 days in FP counseling and methods provision, including DMPA-SC injections. After being deemed competent by the Health Zone authorities, the CHWs participated in campaign days organized at the main marketplaces (Fungurume and Bukenya) of the Health Zone. There, the CHWs installed a stand with signage advertising FP services and the full display of methods they offered. The market radio was also used to advertise the free services. Interested women were individually counseled for FP at chairs set around the stand and for those who were eligible and chose DMPA-SC, the CHWs took them behind a tarp cabin to perform the injection. Women were then invited to come back 3 months later to receive their second injection and given recommendations to visit the local health center in case of side effects. Women who wanted clinical methods, counseled but not offered by the CHWs, were also invited referred to the facility. All contraceptives and supplies (e.g., backpacks, screening and reporting forms) were provided by the local implementing partner for the project. Nurses from referral health facilities were dispatched to supervise the CHWs, ensuring appropriate counseling for the full range of methods and safe injection practices for women receiving DMPA-SC. Each of the two sites (main marketplace of each target Health Zone) organized 2 campaign days per round (initial injection and 3-month follow-up), for a total of 8 campaign days.

### 2.2 Data collection

For both studies, DMPA-SC providers were interviewed at the end of the second round of the pilot (three-month follow-up) by data collectors trained by Tulane University and experienced in digital data collection for FP surveys. Interviewers collected socio-demographic information from CBDs and asked them to assess the training they received. Interviewers also asked the CBDs about their experiences with FP counseling, service provision and DMPA-SC injection, stock-outs, service statistics reporting, and the referral system.

Most questions were similarly formulated across both cadres of providers; this analysis excludes questions specific to certain aspects of a given pilot (e.g., training women for self-injection).

Data were collected using smartphones using the OpenDataKit (ODK) application. Frequencies and Chi2 significance tests were calculated using STATA18 to compare both groups' answers.

## 3 Results

The two cadres of providers (students and CHWs) differed significantly in terms of socio-demographic characteristics (*see* Table 1)*.* As expected, students were younger (25.6 years old on average) than the CHWs (42.3 years old), and they were more educated (only a third of the CHWs had completed high school). Also, the students were less likely to be married or live in union (13.2% for the students) and to have children than the CHWs (73.5%). Students were less likely to be employed (28.3%), compared to the CHWs (52.9% had a job outside of their FP activities). Gender distribution was more equal for the CHWs (47.1% male and 52.9% female) than for the students three quarter of whom (73.6%) were female.

**Table 1:** Sociodemographic characteristics and need for additional training by type of CBD workers, pilot studies for the provision of DMPA-SC at the community level, Democratic Republic of Congo, 2016–7

	Students⁎	Community Health Workers⁎⁎	p Value for two sample *t*-test
	N=53	N=34	
	*Sociodemographic characteristics*		
**Age –** mean	25.6	42.3	.00
**Age –** standard deviation	7.6	9.8	
Female	73.6% *(39)*	52.9% *(18)*	
Male	26.4% (*14)*	47.1% *(16)*	.05
Married or in union	13.2% *(7)*	73.5% *(25)*	.00
Has children	17.0% *(9)*	97.1% *(33)*	.00
Employed	28.3% *(15)*	52.9% *(18)*	.02
			
***What is the highest level of schooling you have ever attended?***			
No schooling	0	0	-
Primary school	0	67.6% (23)	.00
High school⁎⁎⁎	77.4% *(41)*	29.4% (10)	.00
University	22.6% (12)	1 (2.9%)	.00
			
***On which topics would you have liked to receive more training prior to beginning your work as a community-based distributor?***			
Family Planning (FP) methods in general	45.3% *(24)*	**73.5%***(25)*	.01
Injecting women with DMPA-SC	9.4% *(5)*	58.8% *(20)*	.00
Counseling women on side effects	**41.5%***(22)*	44.1% *(15)*	.81
Presenting FP methods to groups	15.1% *(8)*	32.4% *(11)*	.06
Recruiting acceptors	11.3% *(6)*	29.4% *(10)*	.03
Reporting service statistics	15.1% *(8)*	14.7% *(5)*	.96
Referring women to facilities	11.3% *(6)*	20.6% *(7)*	.24
Explaining program to health officials	15.1% *(8)*	17.7% *(6)*	.75

⁎ Medical and nursing schools students.

⁎⁎ Relais Communautaires (community health workers).

⁎⁎⁎ In DRC, Nursing Sciences are part of secondary education / high school curriculum.

Both groups declared that the training had prepared them somewhat or very well to work as CBDs, but the students were slightly less satisfied with the amount of practice they had before starting DMPA-SC injections in the community. Two-thirds of students (67.9%) reported that they received “completely adequate” practice versus 79.4% of CHWs. Differences emerged between the two groups' requests for additional training. About 15.1% of the students felt they needed to be better trained in interacting with Health Zone authorities and clients, and service statistics reporting. However, the most significant differences (p=.00) were requests for additional training performing DMPA-SC injections, mentioned by 58.8% of the CHWs but only 9.4% of the students.

After completing the training, the students and CHWs provided similar FP services (Table 2). The handful of students who did not provide DMPA-SC were either stocked out or had clients who chose other methods, whereas two of the 34 CHWs in Lualaba did not provide DMPA-SC because either they or their clients were not comfortable with lay health workers performing an injection (data not shown in table). Service statistics indicate that, for 2017, the students (not limited to those involved in the self-injection pilot) provided DMPA-SC to 10,801 clients, a number almost equal to those served by facilities and other CBD programs (11,777). In Lualaba, the CHWs provided DMPA-SC to 513 clients (26.4% of all DMPA-SC clients in the two target Health Zones for 2017).^3^

**Table 2:** Activities performed, stock-outs reported, and knowledge of referral system by type of CBD workers, pilot studies for the provision of DMPA-SC at the community level, Democratic Republic of Congo, 2016–7.

	Students	Community Health Workers	
	n=53	n=34	p-Value χ^2^ test for independence
***Which activities did you perform at least once while working as a DMPA-SC community-based provider?***			
**Perform DMPA-SC injection**	**94.3%**	**61.7%**	.00
Group counseling	71.7% *(38)*	85.3% *(29)*	.14
Individual counseling	90.6% *(48)*	64.7% *(22)*	.00
Offer methods other than DMPA-SC	83.0% *(44)*	55.9% *(19)*	.01
Provide referral to health clinics	41.5% *(22)*	26.5% *(9)*	.15

***Which methods were stocked out more than once during the pilot period?***			
Pills	11.3% *(6)*	17.7% *(6)*	.40
Condoms	43.4% *(23)*	23.5% *(8)*	.06
Cyclebeads	18.9% *(10)*	14.7% *(5)*	.62
DMPA-SC	45.3% *(24)*	14.7% *(15)*	.00
			
***What would you say to a woman who preferred a method you do not provide?***			
Refer her to a facility	73.6% *(39)*	**100.0%***(34)*	.00
Insist that she accept DMPA-SC	**5.7%***(3)*	0.0% *(0)*	.16
Insist that she accept another method I provide	**15.1%***(8)*	2.9% *(1)*	.07
Cannot do anything	18.9% *(10)*	0.0% *(0)*	.01
Refer to a facility	75.5% *(40)*	**91.2%***(31)*	.07
Tell her to wait and see if side-effects improve	37.7% *(20)*	20.6% *(7)*	.09
Consult supervisor	0.0% *(0)*	2.9% *(1)*	.21

Most CBDs across groups declared being somewhat or very comfortable with all aspects of FP service provisions at the community level, with 90.4% of the students and 79.4% of the CHWs feeling comfortable performing DMPA-SC injections.

A majority of providers experienced stock-outs: 55.9% of CHWs and 71.2% of students ran out of at least one contraceptive during campaign days. With regards to DMPA-SC, the students were more likely to experience stockouts: 45.3% reported “sometimes or often” stockouts of DMPA-SC compared to 14.7% of CHWs (p=.00).

Students (64.71%) were less likely to report that they received adequate supervision than RECOs (96.3%) (p=.03). Although 82.7% of CHWs, reported receiving individual visits from supervisors, 14.7% of them (n=5) found the supervision “not really” or “not at all” adequate. By contrast, in Lualaba, 91.2% of CHWs said they were supervised in person during their community distribution activities. Nearly all CHWs (93.8%) and 67.44% of the students found the supervision they received to be “very useful”.

Significant differences also appeared between the cadres of providers in their practice of referring women to health facilities. Fewer students (82.7%) reported knowing which structures offered FP services they could refer women to than CHWs (100%) (p=.01). More students (15.1%) than CHWs (2.9%) reported that they would insist that their client accept one of the methods they offered, rather than referring women who wanted a clinical method (IUD, implant) (p=.07). CHWs were more likely to refer women to a facility for management of side effects than the students (Table 2). Overall, only 55.9% of CHWs and 48.1% of students had ever referred a woman to a facility during their activities (p=.48).

When asked about perception of their FP activities in their own communities, students more frequently (76.9%) reported encountering resistances whereas only 41.2% of the CHWs indicated ever hearing open opposition (p=.00). However, all providers reported similar reasons for community resistance with two-thirds of them mentioning rumors surrounding family planning in general (namely increased risks for infertility and cancer incorrectly associated with contraceptive use) rather than their own work as CBDs. *(Data not shown in tables)*.

Finally, almost all providers were “satisfied” or “very satisfied” with their experience and most (100% of students and 88.2% of CHWs) would either “encourage” or “strongly encourage” their peers to work as a community-based FP providers” . Table 3 details the positive and negative aspects reported by both groups.

**Table 3:** Positive and negative aspects of CBDs experience reported, by type of CBD workers, pilot studies for the provision of DMPA-SC at the community level, Democratic Republic of Congo, 2016–7.

	Students	Community Health Workers	p-Value χ^2^ test for independence
	n=53	n=34	
***What were the POSITIVE aspects of your experience as a community-based provider for DMPA-SC?***			
Helping my community	58.5% *(31)*	85.3% *(29)*	.01
Gaining technical experience	71.7% *(38)*	82.4% *(28)*	.26
Improve social skills and client interactions	66.0% *(35)*	44.1% *(15)*	.04
			
***What were the NEGATIVE aspects of your experience as a community-based provider for DMPA-SC?***			
Not enough motivation / financial support	34.0% *(18)*	44.1% *(15)*	.34
Insufficient training / supervision	22.6% *(12)*	29.4% *(10)*	.48
Lack of technical support	35.9% *(19)*	32.4% *(11)*	.74
Too much time commitment	9.4% *(5)*	29.4% *(10)*	.02
Rumors and resistances were frustrating	15.1% *(8)*	2.9% *(1)*	.07

## 4 Discussion

Task-shifting sexual and reproductive health services (and particularly DMPS-SC provision) is a promising strategy for expanding access to contraception in resource-constrained environments [7], [8], [9], but must be tailored to country-specific contexts. In DRC, the provision of DMPA-SC at the community level was initially piloted using medical and nursing school students. The 2017 pilot conducted in Lualaba represents a far more radical effort in task-shifting since the CHWs had not received any formal clinical training before the study. They were also older, had larger families (consistent with the demographics of rural DRC) and operated at marketplaces. Yet the comparison of this group with student providers operating in Kinshasa yielded similar levels of satisfaction with the training they had received, their experiences as CBDs, their motivation to “help their community” and their frustrations with the lack of remuneration and the rumors circulating against FP.

CHWs in Lualaba were initially slightly more intimidated in performing injection, but they eventually reported it to be easy, as was counseling women for other contraceptive methods. The students, while understandably more comfortable with the clinical aspects of DMPA-SC provision, reported being less comfortable with group counseling and recruiting new clients, possibly because they were younger and more intimidated than the Lualaba CHWs, who had been involved in health-related service provision in the community before participating in the DMPA-SC pilot.

Overall, difficulties encountered across all cadres of providers (e.g., frustration with stock-outs or supervision) were more likely to be related to issues beyond their control, rather than to DMPA-SC provision. The prevalence of stock-outs, particularly for the students facing high demand during the campaign days organized in Kinshasa's communities, is a reminder that contraceptive security, from procurement to last mile delivery, is a central issue to successfully scaling-up FP service delivery strategies.

In addition, addressing gaps in the referral system, as providers reported not being completely comfortable on when and how to refer clients to fixed facilities is a key requirement to strengthen the task-shifting process. Keeping the community workers fully integrated and supported within existing health systems is crucial to guaranty the safety and sustainability of such programs [10]. In particular, the fact that the students were more likely to encourage clients to use methods they could provide themselves is problematic for FP quality of care and full contraceptive choice and needs to be addressed through training and supervision [9], [10].

This analysis had several limitations. Providers for both pilots were recruited based on their willingness to participate, their level of education, and their previous performance as either students or CHWs. Thus, these results represent a best-case scenario for task-shifting using these two cadres. In addition, these self-reported data likely reflect some social desirability bias. Finally, while international guidelines and research [11] currently focuses on possibly increased risk for HIV acquisition for women using DMPA-SC, these results were conducted before these were disseminated and could not be included in the community providers training.

## 5 Conclusion

The similarities between the experiences and satisfaction of these two very different groups of providers complements the positive findings from acceptability and feasibility studies among the acceptors of DMPA-SC and key informant interviews among local health authorities.^4^ These results are promising for the on-going scale-up of community-level provision of DMPA-SC by both students and CHWs in DRC.

Following on the 2015 DMPA-SC pilot, the government is now institutionalizing FP training and community-level service provision among nursing schools in the DRC, while other projects are exploring the use of lay health workers to provide DMPA-SC. As these initiatives evolve, it will be necessary to conduct more systematic comparative studies between these different cohorts of providers to assess the quality of FP care delivered and to evaluate strategies for sustaining their commitment and effectiveness.

## Footnotes

1 Estimated at 16.8% for all women and 24.8% for married women in Kinshasa per PMA2020 / R6.

2 See companion article in this supplement.

3 Data extracted from DRC SNIS / DHIS2 database for total number of DMPA-SC Clients served in Kinshasa and Lualaba provinces for 2017.

4 See companion article in this special issue.
